# Two Decades of Outcomes and Quality of Life Following Pencil Beam Scanning Proton Therapy in Children and Adolescents with Rhabdomyosarcoma

**DOI:** 10.3390/cancers17172771

**Published:** 2025-08-26

**Authors:** Dominic Leiser, Tobias Dantonello, Reinhardt Krcek, Leonie Grawehr, Jochen Rössler, Gabriele Calaminus, Damien Charles Weber

**Affiliations:** 1 Center for Proton Therapy, Paul Scherrer Institute, 5232 Villigen, Switzerland; 2 Department of Pediatrics, Division of Pediatric Hematology/Oncology, Inselspital, University of Bern, 3012 Bern, Switzerland; tobias.dantonello@insel.ch (T.D.);; 3 Department of Radiation Oncology, Inselspital, Bern University Hospital, University of Bern, 3012 Bern, Switzerland; 4 Clinic of Pediatric Hematology and Oncology, University Bonn, 53127 Bonn, Germany

**Keywords:** children, adolescents, proton beam radiation therapy, pencil beam scanning proton therapy, rhabdomyosarcoma, quality of life, long term toxicity

## Abstract

Background: RMS is the most common soft tissue sarcoma in children. Pencil beam scanning proton therapy (PBS PT) enables highly conformal dose delivery with reduced exposure to surrounding healthy structures, making it particularly suited for RMS in critical anatomical regions. Long-term clinical outcome data for this new radiation technique are scarce. Purpose: This study reports long-term outcomes and quality of life after PBS PT in children and adolescents with rhabdomyosarcoma (RMS). Methods and Materials: We retrospectively reviewed 114 children and adolescents with RMS (mostly embryonal, *n* = 100; 87.7%) treated between 2000 and 2020. Their median age was 4.6 years (range, 0.3–18). All received systemic chemotherapy according to prospective protocols. The median total PT dose delivered was 52 Gy (RBE; range, 41.4–64.8). Results: After a median follow-up period of 7.1 years (range, 0.3 to 17 years), we observed 26 failures overall; 21 (80.8%) occurred in-field. The 5-year local control and overall survival were 81.2% and 81%, respectively. The composite endpoint (non-ocular grade ≥3 toxicity- and failure-free survival) counting the first occurrence of any failure (local or distant), death, or non-ocular CTCAE v5.0 grade ≥3 toxicity was 77.3% at 5 years. At the start of PT, parents and children reported a quality of life significantly worse than that of a German normative group, but during the follow-up period, their scores improved to normal values in nearly all domains within two years. Conclusions: Our two decades of experience with PBS PT provide data that reflect good local control rates and minimal late non-ocular grade 3 toxicity. We also show that quality of life returned to normal scores in nearly all domains within 2 years. Children and adolescents with RMS seem to benefit from PBS PT in terms of toxicity and quality of life, but further prospective, multi-institutional comparative trials are needed.

## Simple Summary

Children and adolescents with rhabdomyosarcoma often require radiotherapy, which can damage healthy tissues. Pencil beam scanning proton radiotherapy (PBS PT) delivers highly conformal doses while sparing healthy organs. We report our experience over two decades with 114 patients treated between 2000 and 2020 in a single institution. The median observation time was 7.1 years, ranging from 0.3 to 17 years. Five-year local tumor control and overall survival were 81.2% and 81%, respectively. Serious late toxicity was rare: only 16% of patients experienced non-ocular grade ≥3 toxicity. Prospectively collected quality of life questionnaires showed that patients’ quality of life improved over time and returned to normal levels in nearly all domains within two years. These findings suggest that PBS PT offers effective tumor control with limited toxicity, supporting its use in children and adolescents with rhabdomyosarcoma.

## 1. Introduction

Rhabdomyosarcoma (RMS) is the most prevalent soft tissue sarcoma in children and adolescents, accounting for approximately 50% of such sarcomas in this age group and representing about 3–4% of pediatric cancers. It occurs at an incidence of approximately 4.6 cases per million children and adolescents per year [1]. Although chemotherapy remains a cornerstone of treatment, long-term treatment typically requires a multimodal approach that includes surgery and radiotherapy for local control. The prognosis for patients with low- and intermediate-risk disease has improved substantially in recent decades; however, outcomes for those with high-risk or metastatic RMS remain unsatisfactory [2,3]. Local control in anatomically challenging regions—particularly parameningeal and head and neck sites—remains difficult due to the dual imperative of eradicating the disease while preserving critical organ function and developmental integrity.

Over the past three decades, the Center for Proton Therapy at the Paul Scherrer Institute (PSI) has focused on delivering proton therapy (PT), with a particular emphasis on pioneering and applying pencil beam scanning proton therapy (PBS PT) [4]. PBS PT is an advanced form of radiation treatment that delivers protons in narrow, precisely modulated beams layer-by-layer across the tumor volume. PT allows for high conformality in dose delivery while substantially reducing the integral dose to surrounding healthy tissues compared to photon-based techniques, which is particularly important for the pediatric population [5,6]. For more than two decades we have used PBS PT routinely for the treatment of pediatric and adolescent RMS patients. Previous studies have demonstrated favorable clinical outcomes and tolerable toxicity in children treated with PBS PT, achieving good local control and quality of life preservation [7,8,9,10,11,12,13,14,15]. No randomized Phase III studies for this new radiation technique exist and long-term functional and QoL outcomes data in modern PBS cohorts are limited. A recent meta-analysis comparing photon and proton therapy for pediatric RMS reported no significant difference in overall survival but suggested that early local control may be inferior with older proton techniques [16]. The cost-effectiveness of current proton therapy for rhabdomyosarcoma remains unproven; further data for precise modeling are needed [17].

Herein, building on our institutional experience [7,9], we provide updated long-term clinical outcomes in a cohort of children and adolescents with RMS treated with modern PBS PT. In addition to oncologic outcomes and treatment-related toxicity, we report prospectively collected, patient-reported quality of life data, providing a comprehensive view of both disease control and functional impact. By sharing these extensive results, we aim to inform clinical decision making and survivorship care.

## 2. Materials and Methods

### 2.1. Patients

For the period between January 2000 and December 2020, 140 patients with a diagnosis of RMS were identified in our institutional database. For this analysis ten were excluded due to RMS relapse at the time of PBS PT (i.e., PBS PT was the second-line treatment), seven due to receiving a mixed proton/photon treatment, seven due to age over 18 and two due to consent not being provided for the use of their data for scientific purposes (Supplementary Figure S1). Thus, a total of 114 patients were included in the analysis whose characteristics are detailed in Table 1.

All patients received chemotherapy per contemporary protocols (Supplementary Table S1). Alkylating agents/IFO, vincristine, and actinomycin comprised the backbone of all chemotherapy protocols. The median time from diagnosis to PBS PT was 4.6 months (range, 1.3–9.8). Institutional Review Board (IRB) approval was obtained for this study (EKNZ 2021-02192).

### 2.2. PBS PT Treatment

Before 2005, patients were treated using PBS PT at the scanning gantry with energy-degraded beams from a 590 MeV cyclotron, after which time they were treated with a dedicated 250 MeV cyclotron. The proton dose was computed using a dose calculation algorithm developed at PSI [18] or with Eclipse (Varian Medical Systems, Palo Alto, CA, USA). Patients were immobilized using a body cast; for the head and neck, this was accomplished as previously described [9]. Gross tumor volume (GTV) and clinical target volumes were delineated and site-specific constraints were applied as per protocol. Plans were generated to PTV = CTV + 3–5 mm without robust optimization (reflecting historical practice at our center); range and set-up uncertainties were addressed through margins and verification procedures. The dose was prescribed to the mean target dose and reported in Gy (RBE) [Gy (RBE) = proton Gy × 1.1]. The median delivered dose was 52 Gy (RBE) (range 41.4–64.8; Table 1). Only one patient received more than 60 Gy (RBE), due to progression under chemotherapy of high-risk parameningeal RMS. Four low-risk orbital patients received less than 45 Gy (RBE) per protocol. The median number of fractions was 28 (range, 23–36). The dose per fraction was 1.8 Gy (RBE) for 104 patients (91%) and 2 Gy (RBE) per fraction for 9 (8%), in addition to a combination of 1.8/2 Gy (RBE) for one patient (0.8%). PBS PT was delivered in 30 to 77 (median: 40) days.

### 2.3. Follow-Up

Follow-up was organized by the referring physicians per the protocol, and data were collected as previously described [7], by collecting follow-up reports yearly. Acute toxicities were defined as adverse events that occurred between the first day of treatment and 90 days after treatment, late toxicities occurring 90 days or later after PT. All side effects observed were classified according to the National Cancer Institute Common Terminology Criteria for Adverse Events v5.0 grading system (https://evs.nci.nih.gov/ftp1/CTCAE/CTCAE_5.0/CTCAE_v5.0_2017-11-27.xls (accessed on 1 January 2021). Cataracts requiring surgery were graded as G3, but due to the uncomplicated therapy involved, they were later excluded from the toxicity-free survival calculation.

### 2.4. Quality of Life

Health-related quality of life (QoL) was assessed using the validated Pediatric Quality of Life Questionnaire (PedQoL) [19], available in a self- or proxy-report version. This instrument was developed over 20 years ago at the University of Münster in Germany specifically for pediatric oncology patients, can be used for patients over the age of 4 years, and has been validated in different languages. The questionnaire covers eight domains: autonomy (SE); emotional functioning (EV); body image (KB); cognition regarding school and work performances (C); physical functioning in terms of activity, energy, and pain (KV); social functioning with peers (Fr); social functioning with family (Fam); and subjective well-being (Global). For young children (≤4 years), the proxy Pediatric Quality of Life Inventory (PedsQL) was used [20], which covers four scales (physical, emotional, social, and school). The physical, emotional, and social scales were combined into a “psychosocial” scale, and all four scales were combined into a “total” summary scale. The questionnaires were distributed before the start of PBS PT (E1), 2 months after PBS PT (E2), and yearly thereafter (E3 at 1 year, E4 at 2 years, E5 at 3 years, E6 at 4 years). QoL scores were calculated per domain/scale; higher scores suggested a better QoL (ranging from 0 to 100). To compare the QoL values of the study cohort, a normative group comprising healthy children (self-assessment) and caregivers of healthy children (proxy assessment) were included in the analyses [19].

### 2.5. Statistical Analysis

Local control (LC) and overall survival (OS) rates were determined from the PBS PT start date. Death was the event for OS; loco-regional tumor relapse for LC; the composite endpoint (non-ocular grade ≥3 toxicity- and failure-free survival) counted the first occurrence of any failure (local or distant), death, or non-ocular CTCAE v5.0 grade ≥3 toxicity. We estimated survival using Kaplan–Meier curves, compared groups with log-rank tests, and fitted univariable Cox proportional-hazards models to obtain hazard ratios (HRs) with 95% CIs in SPSS Version 28 from (IBM cooperaton, Armonk, NY, USA).

For the PedQoL tool, medians and percentiles were used, and differences between the study cohort and the normative group were tested with the Mann–Whitney U test. For the PedsQL tool, means and standard deviations were used, and differences from the normative group were tested with a *t*-test. No adjustment for multiple testing was performed. Only patients with baseline (E1) data and at least one completed follow-up questionnaire were included in the analysis. Missing data were not imputed.

## 3. Results

### 3.1. Survival and Patterns of Failure

**Figure 1:**
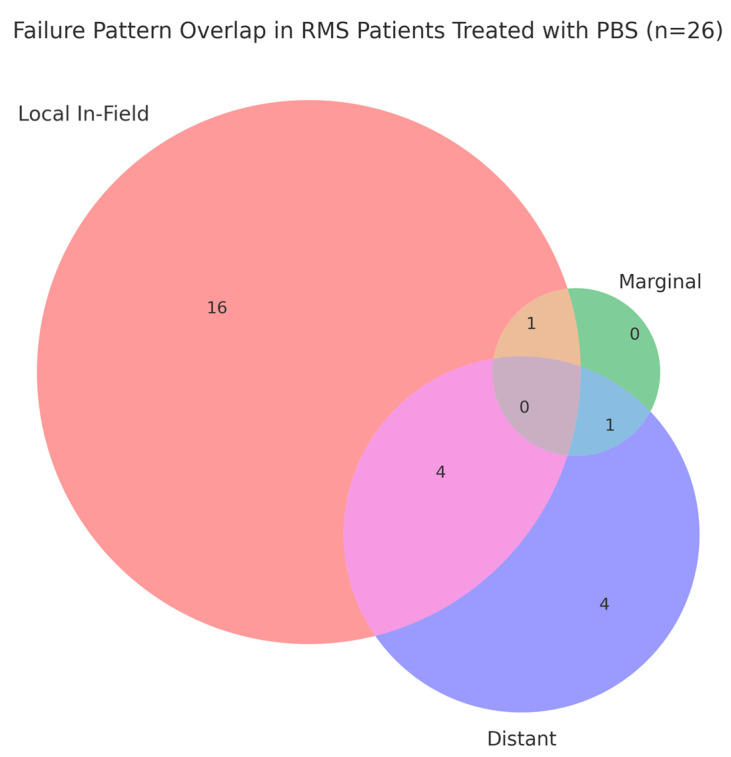
Pattern of failures in 114 children and adolescents treated with proton therapy. Red represents in-field failures, green represents marginal failures, and blue represents distant failures. The remaining colors indicate combinations of these failure types.

After a median follow-up time of 7.1 years (range, 0.3–17), we observed treatment failures in 26 patients in our cohort of 114 children and adolescents. We classified failures in relation to the radiation field/volume. In-field means all of the recurrent tumor received at least 95% of the prescribed dose, marginal means the recurrence was at the edge of the radiation field, specifically within a defined margin of 2 cm, and distant means the recurrent tumor became metastatic. Among the patients with treatment failures, 16 patients experienced in-field recurrences, 4 were distant, 4 combined local infield and distant, 1 involved marginal and distant components, and 1 represented a combination of in-field and marginal failure (Figure 1).

Patients experiencing treatment failure received care in accordance with the standard practices of their local treating physicians. Salvage therapy included chemotherapy for all patients, surgery in 15, and re-irradiation in 6 (including 3 post surgery). Outcomes after salvage were variable: two orbital patients (100%) remained disease-free after surgery/chemotherapy and one parameningeal patient (7%) remained disease-free after receiving all three modalities; the remaining patients all died of progressive disease.

**Figure 2:**
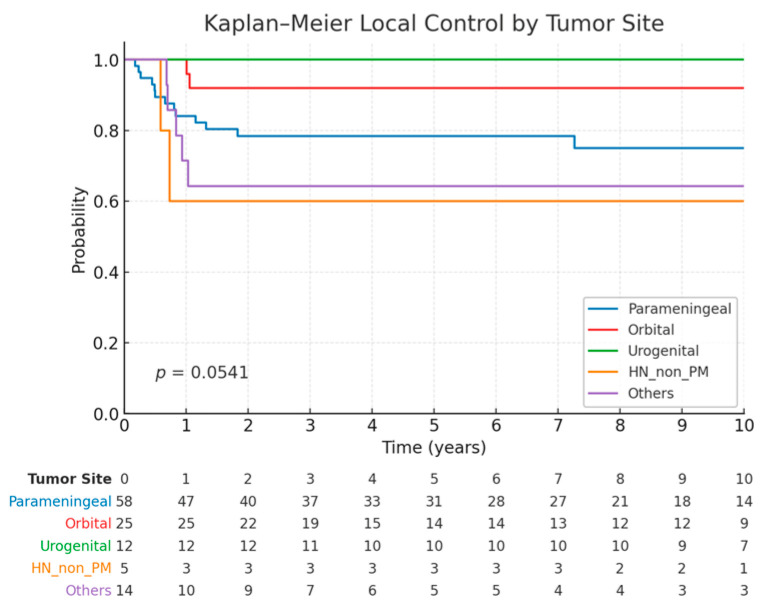
Kaplan–Meier curves of local failure stratified by tumor site. Table below indicate number of patients at risk per year after PBS PT.

The estimated 5-year LC and OS rates were 81.2% (95% CI: 73.8–88.6%) and 81% (95% CI: 73.1–88.3%), respectively. For the parameningeal (PM), orbital, urogenital, head and neck non-PM, and other subgroups, the 5-year LC was 78.4%, 92%, 100%, 60.0%, and 62.5%, respectively (Figure 2). The log-rank value did not reach a statistically significant difference between the groups (*p* = 0.054).

**Figure 3:**
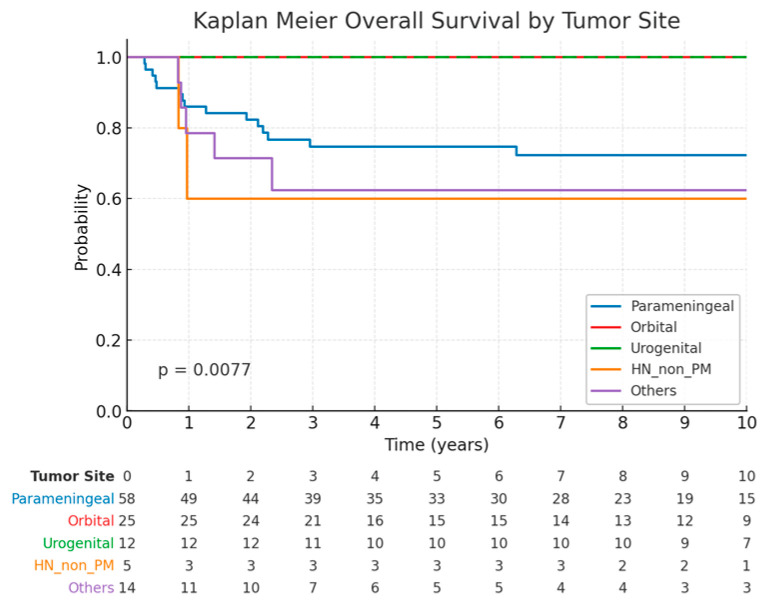
Kaplan–Meier curves of overall survival rates stratified by tumor site. Table above indicates number of patients at risk per year after PBS PT.

The corresponding figures for OS for the parameningeal, orbital, urogenital, head and neck non-parameningeal and other subgroups were 74.1%, 100%, 100%, 60.0%, and 65.2% respectively (Figure 3). Significant differences in OS were observed among the anatomical groups (log-rank *p* = 0.0077).

Additionally, five-year survival by CWS risk subgroup was 100%, 90.9%, 80.4% and 46.2% for the low, intermediate, high, and metastatic groups, respectively (Supplementary Figure S2). Further five-year survival rates and hazard ratios are listed inSupplementary Table S2. In univariable Cox analyses, intracranial extension was associated with inferior LC (HR 2.56, 95% CI 1.11–5.90) and OS (HR 4.28, 1.79–10.20); T2 vs. T1 tumors showed worse LC (HR 3.05, 1.03–9.00) and OS (HR 14.33, 1.93–106.54); and metastasis at diagnosis predicted poorer outcomes (LC HR 5.65, 2.30–13.86; OS HR 5.54, 2.26–13.58).

### 3.2. Toxicity

Table 2 lists the acute and late toxicities, respectively, observed in our 114 RMS patients treated with PBS PT.

PBS PT was well tolerated. In total, only in 19 patients (16.6%) was any type of grade 3 acute toxicity observed. No toxicity higher than grade 3 occurred during or 90 days after treatment.

**Figure 4:**
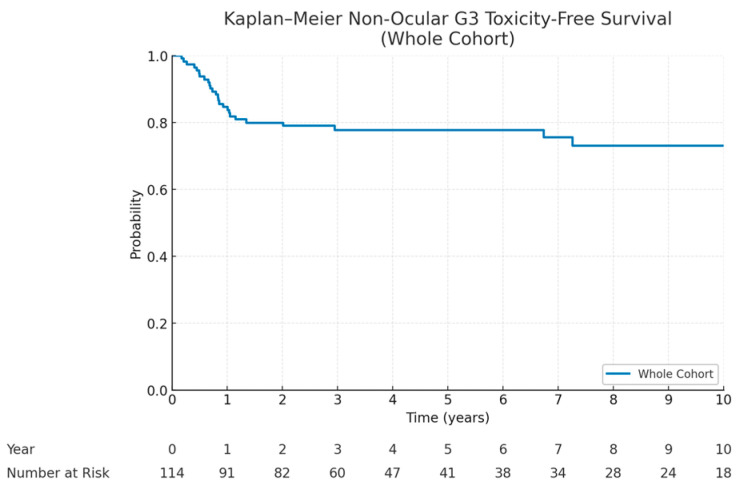
Kaplan–Meier curve of non-ocular grade 3 toxicity-free and failure-free survival. Table above indicates number of patients at risk per year after PBS PT.

The most commonly observed late toxicities were localized alopecia, growth hormone and other endocrinopathies, growth abnormalities, and localized functional impairments. Two secondary radiation-induced tumors were observed 51.6 months and 64 months after PT. For visual toxicity, cataracts treated by surgery were counted as G3, resulting in 76% (*n* = 19/25) of orbital RMS patients being listed under this grade. One patient developed non-cataract related blindness as a late toxicity. As cataracts are readily manageable with surgical intervention, we focused our analysis on grade 3 non-ocular toxicities, for which the 5-year toxicity-free survival rate was 77.3% (95% CI: 69.1–85.5%) (Figure 4).

No significant univariable predictors of late non-ocular grade ≥3 toxicity were identified (Supplementary Table S3).

### 3.3. Quality of Life Results

**Figure 5:**
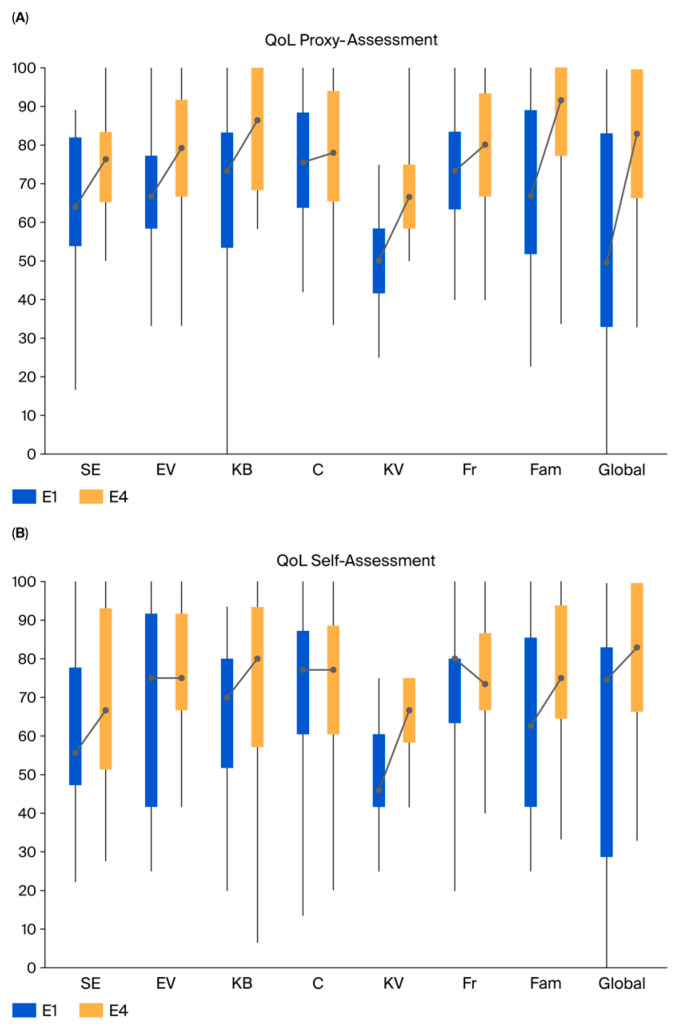
Quality of life assessment. (**A**) By parents at the beginning of the proton therapy (E1) and two years after (E4). (**B**) Children’s self-assessment. Legend: autonomy (SE); emotional functioning (EV); body image (KB); cognition regarding school and work performances (C); physical functioning in terms of activity, energy, and pain (KV); social functioning with peers (Fr); social functioning with family (Fam); and subjective well-being (Global). Boxes show the interquartile range (25th–75th percentiles); whiskers denote the full range (minimum–maximum), dots indicate the median, the line within each box the trend E1 to E4.

Sixty-five patients were eligible for QoL evaluation (Supplementary Table S6). Before and during PT, children and their caregivers reported significantly worse ratings than the normative group. However, QoL improved over the follow-up period, approaching normal values in nearly all domains two years after PT (Figure 5, Supplementry Firgure S3). At the start of proton therapy (E1), parents rated their children’s QoL as significantly worse than the normative group in all domains except for “autonomy” and “cognition”. At E4, there were no longer significant differences between the two groups (Mann–Whitney U test) (Figure 5A, Supplementary Table S3). At E1, the children gave almost similar QoL ratings to the normative group, with slightly better ones for “cognition” and significantly better ones for “body image”. The domains “physical functioning”, “global function”, and “social functioning—family” show negative significant differences compared to the normative group. At E4, the difference in the “physical functioning” domain changed: patients rated their body image higher compared to their ratings at E1 and the normative group. In addition, even though no further significant differences emerged, except for “social functioning—friends”, the children consistently rated all other domains higher or at the normative level (Figure 5B, Supplementary Table S4). In addition, 3 years after the end of proton therapy (E5), the self-reported QoL scores in all domains except “social functioning—friends” were better than at the beginning of therapy (E1). (Supplementary Table S4). Temporal trends over all time points and comparison to the normative group are shown in Supplementary Figure S3. Comparable effects were also observed in PedsQL scores in children aged ≤ 4 (Supplementary Table S5).

## 4. Discussion

This study presents the most comprehensive single-institution analyses to date of children and adolescents with RMS treated with PBS PT [16], with long-term follow-up, stratified survival outcomes by tumor site, and prospectively assessed quality of life (QoL). Our findings demonstrate that PBS PT-based treatment achieves disease control and toxicity profiles that seem to be superior to those in historical photon-based treatments, particularly in anatomically complex or high-risk subgroups. However, these outcomes must be interpreted within the context of key limitations related to cohort composition and the absence of a randomized comparator, e.g., referral bias which is also affected by socioeconomic status. We do not expect a large confounding effect due to different protocols as there has been no significant progress in the systemic treatment of RMS in recent decades. The backbone of treatment remains alkylating agents/IFO, vincristine, and actinomycin. Other drugs have been introduced and most have since been discontinued. Maintenance therapy has brought marginal improvements. The differences between chemotherapy protocols are, therefore, not that large. Our cohort had localized tumors and the focus of this study was primarily on local control, therefore we do not expect a large confounding effect. The observed 5-year LC and OS rates of 81.2% and 81%, respectively, are in line with previously published cooperative group data for intermediate- and high-risk RMS, including those from the EpSSG, COG, and IRS studies [3,16,21,22,23,24]. Particularly noteworthy is the 5-year LC of 78.4% for PM RMS—the largest subgroup in our cohort—which has historically been associated with poor local control due to intracranial extension, cranial nerve involvement, and challenges in delivering curative radiation doses [25]. The favorable outcomes achieved here suggest that PBS PT, with its capacity for high-dose conformity and superior sparing of adjacent organs at risk, can facilitate optimal target coverage without compromising local control, even in these challenging sites. Photon IMRT series report ≥grade 3 late toxicities in ~33% of head and neck RMS patients [26], higher than our 16% overall rate. Of note are the 5-year LC and OS rates in our cohort—which seem to exceed those reported in photon-based series [16]—especially considering the high proportion of patients with PM tumors (50.9%) and unfavorable anatomical locations (68.4%). LC was highest in orbital and urogenital RMS (92% and 100%) [27] and remained acceptable in PM RMS (78.4%), a subgroup historically associated with higher rates of failure. Of note, in our PM cohort, 40 (69%) presented with the additional risk factor of intracranial extension [28], which was associated with worse outcome in univariate analysis. The lowest LC and OS were observed in non-PM head and neck and “other” anatomical locations (e.g. LC: 60.7% and 62.5%), consistent with the known challenges of treating these tumors due to their proximity to critical structures.

The failure analysis in relation to the radiation volume showed that relapses were predominantly in-field (*n* = 21; 80%); this distribution does not suggest a marginal miss pattern attributable to the sharp lateral penumbra of PBS PT. These findings are consistent with biological resistance and are in line with prior reports from our institution and others using proton therapy in pediatric RMS [28,29,30].

In terms of toxicity, the rate of grade ≥3 non-ocular late toxicities remained low, with a 5-year toxicity and relapse-free survival of 80.7%. Most events were related to endocrinopathies, growth abnormalities, or localized functional impairments, and severe complications were infrequent. Only two radiation-induced secondary malignancies were observed. This favorable toxicity profile correlates with good QoL after therapy. Overall, this supports the tissue-sparing advantage of PBS, particularly in younger patients with a long life expectancy and heightened vulnerability to late effects.

A unique aspect of our study is the prospective assessment of QoL using validated instruments. At the initiation of PT, both patients and caregivers reported QoL as significantly worse than healthy controls. However, across follow-up assessments, patients showed a consistent and meaningful improvement in most domains. By 2–3 years post-treatment, both self- and proxy-assessed QoL scores were comparable to or better than those of the normative group in all domains except social functioning with peers. We hypothesize that residual deficits in peer social functioning are usually related to long hospitalization and reintegration often not into the same peer group as before (school, new class). We recommend fast re-integrating, continued schooling during treatment as far as possible (schooling by teachers at home or via online avatar), maintaining contact with friends, and returning to kindergarten/school quickly with the same caregivers as before if possible, as well as rehabilitation and peer support programs as survivorship care.

Several limitations of this study must be acknowledged. The retrospective design limits the completeness of data, particularly for long-term cognitive and growth abnormality assessments. The absence of formal robust optimization is acknowledged as a limitation; however, the predominantly in-field failure pattern (21/26) argues against systematic marginal miss or range uncertainty as a principal cause of recurrence. Despite this study involving the largest single-institution cohort treated with PBS PT for RMS, the sample size of some subgroups (e.g., head and neck non-PM, “other” sites) was small, which may affect the statistical power and generalizability of the subgroup analyses. Furthermore, the cultural and linguistic diversity in our patient population, which comprised many different nationalities, may have influenced the QoL assessments, perhaps in contrast to the normative group, which was sampled from Germany. In addition, while the age distribution was not significantly different in the PedsQL groups, it was in the PEDQoL groups: a higher proportion of children were younger in the patient group than in the normative group, and 17- and 18-year-olds were not represented at all.

Nonetheless, this study provides the most comprehensive long-term evaluation of PBS PT for RMS to date. Our results support the integration of PBS PT into multimodal RMS treatment, especially for anatomically challenging or high-risk tumors. Other radiation modalities are brachytherapy and stereotactic radiosurgery. Considering the high cost and limited availability of proton therapy, cost-effectiveness analyses and policies promoting equitable access are warranted. Ongoing international trials will help further define the role of modern radiotherapy techniques within risk-adapted therapeutic strategies.

## 5. Conclusions

In conclusion, our two-decade experience with PBS PT in children and adolescents with RMS indicates good local control and survival outcomes. Furthermore, patient-reported QoL normalized 2 years post-therapy, underscoring the potential of PBS PT as an advantageous treatment modality for this patient population with low late toxicity rates. Further studies regarding growth abnormalities, e.g., facial hypoplasia, should be performed, which are currently being undertaken by our group for orbital RMS. Studies with longer endocrine follow-up are also needed. Future prospective, multi-institutional trials with standardized late-effect and quality of life endpoints are essential to further validate the long-term benefits of PBS PT and guide risk-adapted treatment strategies.

## Figures and Tables

**Table 1:** Patient and tumor characteristics of the 114 children and adolescents.

	Absolute Number	%
Demographics		
Gender, male	60	52.6%
Gender, female	54	47.4%
Age at start of PBS PT		
Age, mean	5.8	
Age, median	4.6	
Age, max	18.0	
Age, min	0.3	
Age >10	21	18.4%
Time from first diagnosis to PBS PT		
Mean, days	138	
Min time until PBS PT, days	40	
Max time until PBS PT, days	807	
Histology of disease		
Embryonal	100	87.7%
Alveolar (FOXO1 status unknown)	3	2.6%
Alveolar (FOXO1 translocation positive)	8	7.0%
Alveolar (FOXO1 translocation negative)	3	2.6%
Tumor site		
Parameningeal HN-PM	58	50.9%
Orbit (ORB)	25	21.9%
Urogenital, bladder/prostate (UG-BP)	6	5.3%
Head and neck, non-parameningeal (HN-nPM)	5	4.4%
Urogenital, non-bladder/prostate (UG-nBP)	6	5.3%
Extremity (EXT)	3	2.6%
Others (OTH)	11	9.7%
Favorable vs. unfavorable location		
Favorable	36	31.6%
Unfavorable	78	68.4%
Intracranial extension		
Yes	40	35.1%
No	74	64.9%
Intergroup RMS Study Group		
Ia	1	0.9%
Ib	0	
IIa	9	7.9%
IIb	1	0.9%
IIc	0	
IIIa	79	69.3%
IIIb	11	9.7%
IV	13	11.4%
Modified Oberlin Prognostic Score		
Unfavorable metastatic disease (2–4 adverse factors)	2	
Favorable metastatic disease (0–1 adverse factors)	11	
T Stage		
T1a	32	28.1%
T1b	3	2.6%
T2a	25	21.9%
T2b	54	47.4%
Positive lymph nodes at diagnosis		
Yes	17	14.9%
No	97	85.1%
Metastases at diagnosis		
Yes	13	11.4%
No	101	88.6%
Location of metastases		
None	101	88.6%
Lung	11	0.7%
Bone	1	0.9%
Ascites	1	0.9%
Size at diagnosis >5 cm	57	50.9%
PBS PT treatment		
Days under PBS PT		
Mean	40	
Median	40	
Min	30	
Max	77	
Number of fractions		
Median	28	
Min	23	
Max	36	
Total dose in Gy (RBE)		
Mean	51.9	
Median	52	
Min	41.4	
Max	64.8	
Treatment details		
Anesthesia during PBS PT	78	68.4%
Chemotherapy prior to PBS PT	114	100.0%
Chemotherapy concomitant to PBS PT	98	86.0%
Macroscopic residual tumor before PBS PT	96	84.2%

Abbreviations: PBS PT: pencil beam scanning proton therapy, FOXO1 PAX3-FOXO1 fusion gene. IRS group: intergroup rhabdomyosarcoma study clinical grouping: I = R0, N0, IIa = R1-N0, IIb = R1-N1resected, IIc = R1-N1resected distal involved, IIIa = R2 after biopsy, IIIb = R2 after major resection (>50%), IV = metastasis at dx. Modified Oberlin Prognostic Score: (1 point for each adverse factor): age ≥10 y, extremity, other, unidentified primary site, bone and/or bone marrow involvement, ≥3 metastatic sites. T stage: T1: confined to the anatomic site of origin, a: ≤5 cm in diameter, b: >5 cm in diameter; T2: extension and/or fixative to the surrounding tissue, a: ≤5 cm in diameter, b: >5 cm in diameter. Number of fractions: number of proton treatments. Gy (RBE)**:** treatment dose (Gy in relative biological effectiveness to photons).

**Table 2:** Acute and late toxicities observed in 114 children and adolescents with RMS treated with PBS PT.

	**Type of Toxicity**	**PM RMS** (*n* = 58)	**Head and Neck non-PM RMS:** (*n* = 5)	**Orbital RMS:** (*n* = 25)	**UG RMS** (*n* = 12)	**Other RMS** (*n* = 14)
		**Any Grade***n* (%) G3 * *n* (%)	Any Grade *n* (%) G3 * *n* (%)	Any Grade *n* (%) G3 * *n* (%)	Any Grade *n* (%) G3 * *n* (%)	Any Grade *n* (%) G3 * *n* (%)
Acute	Dermatitis	54 (93%) 3 (5%)	4 (80%) 2 (40%)	23 (92%) 0	11 (91%) 0	13 (92%) 1 (7%)
	Mucositis	48 (83%) 11 (19%)	4 (80%) 2 (40%)	12 (48%) 0	7 (58%) 1 (8%)	4 (29%) 0
Late	Localized alopecia	13 (22%) 0	0 0	1 (4%) 0	0 0	1 (7%) 0
	Decreased growth velocity	7 (12%) 4 (6%)	0 0	0 0	0 0	0 0
	Growth hormone deficiency	18 (31%) 0	0 0	3 (12%) 0	0 0	1 (7%) 1 (7%)
	Other endocrinopathies	18 (31%) 0	1 (20%) 0	3 (12%) 0	2 (16%) 1 (8%)	1 (7%) 0
	Facial hypoplasia	18 (31%) 2 (3.4%)	1 (20%) 0	9 (36%) 1 (4%)	0 0	0 0
	Visual complications	18 (31%) 8 (14%)	0 0	21 (84%) 19 (76%)	0 0	0 0
	Hearing impairment	13 (22%) 5 (8%)	0 0	0 0	1 (8%) 0	0 0
	Dental growth impairment	8 (13%) 0	0 0	0 0	0 0	0 0
	Chronic nasal and sinus congestion	3 (5%) 0	0 0	0 0	0 0	0 0
	Urinary complication	0 0	0 0	0 0	4 (33%) 0	3 (21%) 1 (7%)
	Defecation problems	0 0	0 0	0 0	2 (16%) 0	1 (7%) 0
	Secondary cancer (radiation-induced)	1 (1.7%) 1 (1.7%)	0 0	0 0	0 0	1 (7%) 1 (7%)

* According to CTCAE ver.5.0. toxicity grading: Grade 1 Mild; asymptomatic or mild symptoms; clinical or diagnostic observations only; intervention not indicated. Grade 2 Moderate; minimal, local or noninvasive intervention indicated; limiting age-appropriate instrumental ADL. Grade 3 Severe or medically significant but not immediately life-threatening; hospitalization or prolongation of hospitalization indicated; disabling; limiting self-care. Grade 4 Life-threatening consequences; urgent intervention indicated. Grade 5 Death. Cataract surgery is graded as CTCAE v5.0 grade 3 but excluded from the composite endpoint of non-ocular grade ≥3 toxicity- and failure-free survival. Abbreviations: *n* = number RMS: rhabdomyosarcoma; PM: parameningeal RMS; UG: urogenital.

## Data Availability

The data of this study are available via the corresponding author upon reasonable request.

## Supplementary Materials

The following supporting information can be downloaded at https://www.mdpi.com/article/10.3390/cancers17172771/s1. Supplementary Figure S1. CONSORT Flow diagram. Supplementary Table S1. Treatment protocol table. Supplementary Table S2. Tabulated 5-Year LC, OS Tox free Survival rates and univariate analysis. Supplementary Figure S2. Kaplan–Meier curve stratified by CWS subgroup. Supplementary Table S3. PEDQoL Proxy-Reported Quality of Life Scores by Domain. Supplementary Table S4. PEDQoL Self-Reported Quality of Life Scores by Domain. Supplementary Table S5. Pediatric Quality of Life Inventory (PedsQL) scores by Domain. Supplementary Figure S3. Temporal trends of QoL per Domain. Supplementary Table S6. Patient QoL Questionnaire Return Rates and Reasons for Non-Participation.
