# Supplementary material for: Two Decades of Outcomes and Quality of Life Following Pencil Beam Scanning Proton Therapy in Children and Adolescents with Rhabdomyosarcoma
Source: Cancers (Basel). 2025 Aug 26;17(17):2771. doi: 10.3390/cancers17172771 (PMC12427509; doi:10.3390/cancers17172771)
Supplement: Supplementary file 1 [file cancers-17-02771-s001.zip › cancers-3760136-supplementary.pdf]

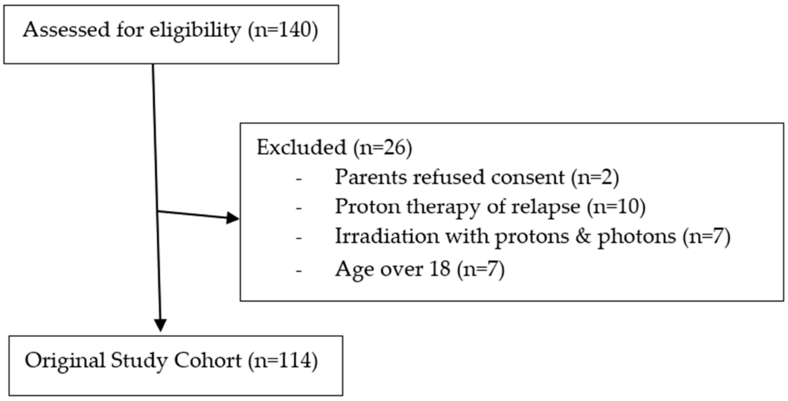

Figure S1. Flow diagram of patient inclusion.

Table S1. Treatment protocols table. Patients were treated according this established protocols, which guided both chemotherapy administration and overall patient management.

**Treatment Protocol**		
CWS Guidance	78	68.4%
EpSSG RMS 2005	12	10.5%
EpSSG/MRSTS05	9	7.9%
MMT-95	3	2.6%
COG-ARST 0531	3	2.6%
COG D8903	1	0.9%
SIOP	1	0.9%
MSKCC 03-099A	1	0.9%

Table S2. Five-year outcomes and univariable hazard ratios for key clinical factors. For each demographic or clinical factor, the table lists the 5-year local control (LC), overall survival (OS) and non-ocular grade ⩾ 3 toxicity-free survival (Tox-free) rates (expressed as percentages) for each category, followed by the hazard ratio (HR) and its p-value (P) relative to the reference category (shown in the first row of each factor). Abbreviations: HN_non-PM = non-parameningeal head and neck; T1/T2 = tumour size categories (<5 cm vs >5 cm); GU = urogenital; IRS = Intergroup Rhabdomyosarcoma Study (risk groups I–IV, where higher Roman numerals denote higher risk).

Factor / Group	5-year local contorl			5-year overall survival			5 yEAR G3 Tox free SURVIVAL		
	(%)	HR (95 % CI)	P	(%)	HR (95 % CI)	P	(%)	HR (95 % CI)	P
**Sex**									
Female (ref.)	81.5	–	–	81.5	–	–	77.4	–	–
Male	81.7	1.05 (0.46–2.44)	0.903	81.7	0.98 (0.42–2.28)	0.969	79.7	0.83 (0.41–1.70)	0.608
**Age**									
≤ 10 yr (ref.)	79.3	–	–	80.4	–	–	76.7	–	–
> 10 yr	90.9	0.58 (0.17–1.97)	0.385	86.4	0.59 (0.17–1.99)	0.393	86.4	0.38 (0.11–1.25)	0.111
**Intracranial extension**									
No (ref.)	86.5	–	–	89.2	–	–	86.3	–	–
Yes	72.5	2.56 (1.11–5.90)	**0.028**	67.5	4.28 (1.79–10.20)	**0.001**	64.1	2.93 (1.42–6.04)	0.004
**T-stage**									
T1 (ref.)	91.4	–	–	97.1	–	–	91.4	–	–
T2	77.2	3.05 (1.03–9.00)	**0.044**	74.7	14.33 (1.93–106.54)	**0.009**	72.7	4.03 (1.22–13.27)	0.022
**Lymph-node status**									
Negative (ref.)	81.4	–	–	80.4	–	–	77.3	–	–
Positive	82.4	1.01 (0.30–3.41)	0.987	88.2	1.01 (0.30–3.40)	0.991	86.7	0.67 (0.26–1.76)	0.422
**Metastasis at diagnosis**									

Non-metastatic (ref.)	86.1	–	–	86.1	–	–	83.8	–	–
Metastatic	46.2	5.65 (2.30–13.86)	**< 0.001**	46.2	5.54 (2.26–13.58)	**< 0.001**	38.5	4.58 (2.04–10.28)	< 0.001
**Histology**									
Embryonal (ref.)	82.0	–	–	83.0	–	–	79.6	–	–
Alveolar	78.6	1.11 (0.33–3.76)	0.863	71.4	1.65 (0.56–4.86)	0.368	71.4	1.21 (0.42–3.47)	0.722
**Total dose**									
≤ 52 Gy (ref.)	83.1	–	–	84.7	–	–	83.1	–	–
> 52 Gy	80.0	1.47 (0.64–3.41)	0.364	78.2	1.72 (0.73–4.02)	0.211	73.6	1.50 (0.72–3.10)	0.280
**Tumour site**									
Parameningeal (ref.)	78.4	–	–	74.1	–	–	73.2	–	–
Orbital	92.0	0.27 (0.06–1.18)	0.082	100	0.00	–	96.0	0.20 (0.03–1.47)	0.112
Urogenital	100.0	0.00	–	100	0.00	–	100.0	0.13 (0.02–0.94)	0.043
HN_non_PM	60.0	1.66 (0.37–7.36)	0.504	60.0	1.52 (0.35–6.64)	0.579	60.0	0.92 (0.22–3.96)	0.916
Others	62.5	1.94 (0.69–5.43)	0.209	65.2	1.74 (0.63–4.79)	0.283	57.1	1.44 (0.58–3.59)	0.431
**IRS risk group**									
I/II (ref.)	90.9	–	–	90.9	–	–	90.9	–	–
III	85.6	2.03 (0.27–15.45)	0.494	85.6	1.93 (0.25–14.66)	0.526	83.0	2.86 (0.39–21.30)	0.304
IV	46.2	10.74 (1.32–87.28)	**0.026**	46.2	10.05 (1.24–81.72)	**0.031**	38.5	12.00 (1.50–95.93)	0.019

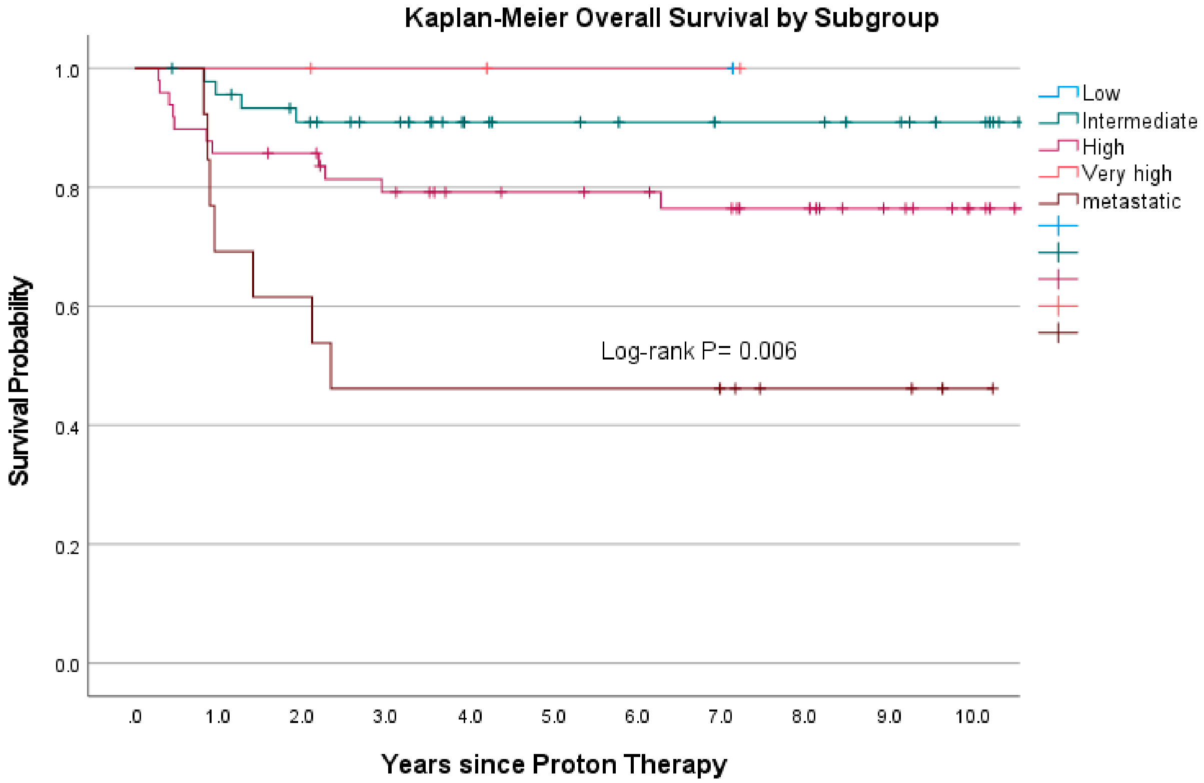

Group/Year	0	1	2	3	4	5	6	7	8	9	10
Low	1	1	1	1	1	1	1	1	0	0	0
Intermediate	46	43	39	35	28	26	24	23	23	21	18
High	51	44	42	37	33	32	31	29	26	21	16
Very High	3	3	3	2	2	1	1	1	0	0	0
Metastatic	13	9	8	6	6	6	6	5	3	3	1

Figure S2. Kaplan–Meier curves of overall survival rates stratified Subgroup. Significant differences in OS were observed among the subgroups (log-rank p = 0.006). Subgroup “Low” showed the highest survival rates, whereas “metastatic” group had the poorest outcomes.

Table S3. **PEDQoL Proxy-Reported Quality of Life Scores by Domain.** Parental proxy assessments using the Pediatric Quality of Life Questionnaire (PEDQoL) are reported across the eight domains; autonomy (SE); emotional functioning (EV); body image (KB); cognition regarding school and work performances (LV); physical functioning in terms of activity, energy, and pain (KV); social functioning with peers (SV_F); social functioning with family (SV_F); and subjective well-being (AF). Scores reflect the caregivers' perception of the child's quality of life on a scale from 0 to 100 (higher scores indicate better QoL). Time points are described below.

PEDQOL PROXY Normgroup (children: 4- 18 yrs) and RMS patients

	SE	EV	KB	LV	KV	SF_F	SV_Fa	AF
	N<br>Median	N<br>Median	N<br>Median	N<br>Median	N<br>Median	N<br>Median	N<br>Median	N<br>Median
Norm	190	232	230	230	231	232	232	231
	66,67	75,00	86,67	80,00	66,67	80,00	83,33	83,33
E1	20	40	39	41	39	37	40	37
	61,11	66,67	73,33	75,00	50,00	73,33	66,67	50,00
E2	18	39	38	39	38	38	38	37
	61,11	75,00	73,33	66,67	58,33	70,83	75,00	66,67
E3	23	44	44	45	46	44	44	42
	77,78	75,00	83,33	80,00	66,67	80,00	80,56	83,33
E4	22	42	40	42	42	41	42	42
	74,17	79,17	86,67	77,50	66,67	80,00	91,67	83,33
E5	21	40	39	40	40	40	40	40
	73,33	79,17	83,33	83,33	66,67	75,00	88,89	83,33
E6	27	39	39	39	39	39	39	39
	77,78	75,00	86,67	75,00	66,67	75,00	83,33	83,33

Table S4. PEDQoL Self-Reported Quality of Life Scores by Domain (E1–E5).

Self-reported scores from the Pediatric Quality of Life Questionnaire (PEDQoL) are presented across the same eight domains: Assessments were performed at standardized time points: start of PBS PT (E1), 2 months after PBS PT (E2), and yearly thereafter (E3 at 1 year, E4 at 2 years, E5 at 3 years, E6 at 4 years). Scores range from 0 to 100, with higher values indicating better perceived quality of life. Discrepancies between proxy and self-assessments are noted and may reflect differential perception, especially in psychosocial domains. Normative data are shown in the first line.

PEDQOL SELF Normgroup (children: 4- 18 yrs) and RMS patients

	SE	EV	KB	LV	KV	SF_F	SV_Fa	AF
	N<br>Median	N<br>Median	N<br>Median	N<br>Median	N<br>Median	N<br>Median	N<br>Median	N<br>Median
Norm	793	794	785	793	794	791	791	789
	66,67	75,00	66,67	66,67	58,33	80,00	75,00	83,33
E1	21	22	22	22	22	22	22	22
	55,56	75,00	70,00	76,67	45,83	80,00	62,50	75,00
E2	21	21	20	21	21	21	21	21
	72,22	83,33	73,33	80,00	50,00	73,33	66,67	66,67
E3	20	22	21	22	22	22	22	21
	72,22	87,50	86,67	80,00	66,67	76,67	66,67	83,33
E4	20	22	22	22	22	22	22	22
	66,67	75,00	80,00	76,67	66,67	73,33	75,00	83,33
E5	22	28	28	28	28	28	28	28
	72,22	83,33	81,67	80,00	66,67	77,50	79,17	83,33
E6	25	30	30	30	30	30	30	30
	72,22	83,33	80,00	77,50	70,83	74,17	77,78	83,33

Table S5. Pediatric Quality of Life Inventory (PedsQL) scores from self-reporting patients aged ≤4 years are presented across the physical, emotional, and social functioning domains. Data are shown at five evaluation time points (E1–E4), corresponding to baseline, the end of treatment, and long-term follow-up intervals. The scores range from 0 to 100, with higher values denoting better quality of life.

PedsQL Normgroup and RMS proxy group with mean

		Physical	Emotion	Social	School	Psychosocial	Totalsum
		N<br>**Mean**<br>SD	N<br>**Mean**<br>SD	N<br>**Mean**<br>SD	N<br>**Mean**<br>SD	N<br>**Mean**<br>SD	N<br>**Mean**<br>SD
Norm	N	2882	2883	2881	1407	2872	2862
	**Mean**	**89,80**	**84,26**	**88,41**	**87,80**	**86,49**	**87,84**
	SD	25	19	20	23	16	18
**E1**	N	20	21	20	6	20	20
	**Mean**	**80,20**	**67,64**	**89,50**	**45,83**	**77,44**	**78,59**
	SD	14,97	17,29	10,37	29,23	12,38	12,01
**E2**	N	17	17	17	7	17	17
	**Mean**	**79,96**	**74,56**	**86,18**	**53,57**	**77,90**	**78,52**
	SD	17,57	14,95	12,31	33,97	13,29	12,98
**E3**	N	11	11	11	8	11	11
	**Mean**	**83,81**	**71,82**	**85,45**	**73,96**	**77,64**	**80,12**
	SD	9,36	9,56	14,91	16,93	12,41	9,71
**E4**	N	7	7	7	5	7	7
	**Mean**	**80,55**	**71,43**	**87,14**	**73,33**	**78,76**	**79,38**
	SD	12,67	18,19	9,94	19,00	13,49	12,68

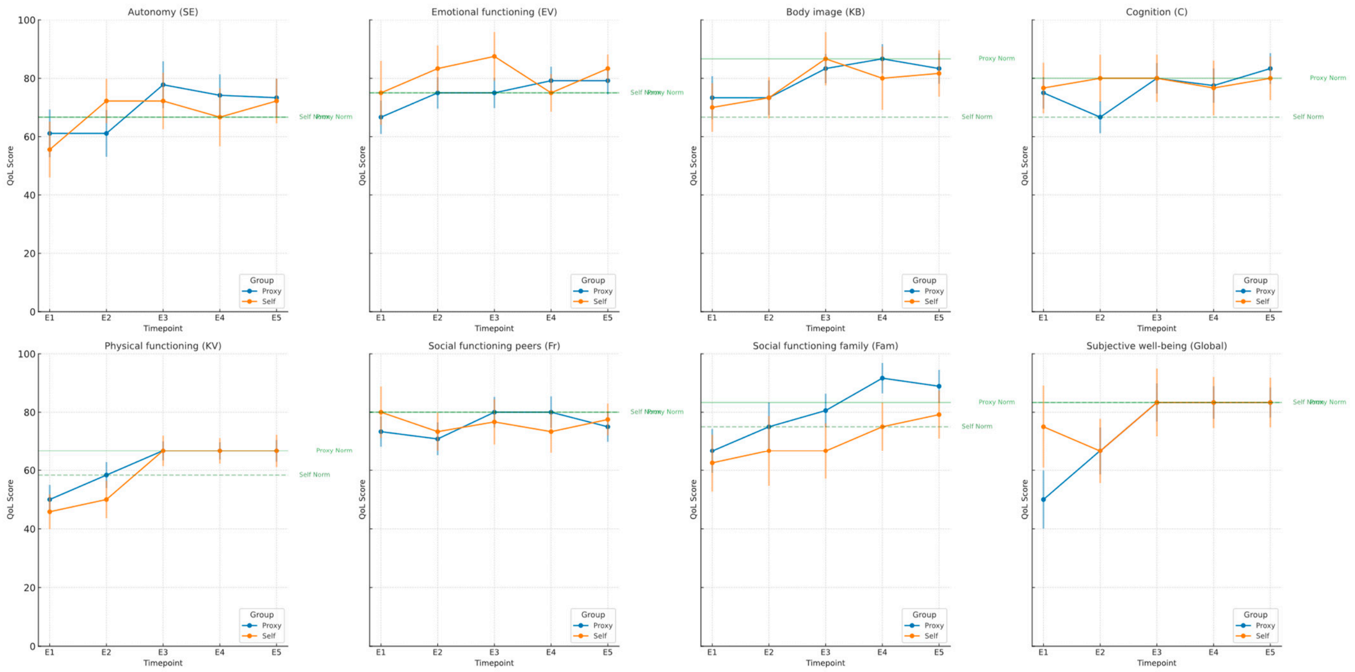

Figure S3. Temporal trends in health-related quality of life (QoL) domains across five time points (E1–E5) for children and adolescents aged 5–18 years, assessed via self-report (“Self”) and parent proxy-report (“Proxy”).
Each panel represents a distinct QoL domain. Median values are plotted for each group, with vertical whiskers indicating the 95% confidence intervals. Normative values derived from a reference population are displayed as faint horizontal lines: green solid line (Proxy Norm), green dashed line (Self Norm).

Table S6. Patient Questionnaire Return Rates and Reasons for Non-Participation.

Time Point	Eligible Patients	Patients Who Returned Questionnaire	Deceased	Recurrence	Time Point Not Yet Reached	Lost to Follow-Up	Too Old
E1	65	65 (100%)	0	0	0	0	0
E2	65	56 (86%)	2	2	0	0	0
E3	61	54 (89%)	2	0	0	0	1
E4	58	50 (86%)	0	0	1	0	1
E5	56	43 (77%)	0	0	1	2	1
E6	52	41 (79%)	0	1	0	4	0
